# Descriptive Study to Assess the Correlation Between Patterns of Dietary Consumption With Systolic and Diastolic Blood Pressure and Random Blood Sugar Among Adults With Diabetes at the Rural and Urban Communities of Uttarakhand

**DOI:** 10.7759/cureus.95815

**Published:** 2025-10-31

**Authors:** Murali Mohanam, Sonia, Neetu Kataria, Devnarayan, Vasantha Kalyani

**Affiliations:** 1 Nursing, All India Institute of Medical Sciences, Rishikesh, Rishikesh, IND; 2 Nursing, All India Institute of Medical Sciences, Raebareli, Raebareli, IND; 3 Nursing, Indira Gandhi National Tribal University, Amarkantak, IND; 4 Nursing, All India Institute of Medical Sciences Deoghar, Devipur, IND

**Keywords:** blood pressure, diabetes mellitus, diastolic blood pressure (dbp), dietary pattern, diet correlated with blood sugar and blood pressure, random blood sugar (rbs), systolic blood pressure (sbp), type 2 diabetes

## Abstract

Background

India leads the world with many patients with diabetes, so it is termed the “diabetes capital of the world”. The aim of the study was to find a correlation between the dietary consumption patterns and systolic blood pressure (SBP), diastolic blood pressure (DBP), and random blood sugar (RBS) among adults with diabetes.

Method

A descriptive-correlational research design was used to collect data regarding dietary consumption among 159 adults with diabetes via a semi-structured questionnaire, and their physiological parameters were measured (SBP, DBP, and RBS).

Results

The mean age of the participants was 53.4±1.23 years. The majority of them were female participants (n=87; 54.7%) and were living in a joint family (n=87; 54.7%). The mean dietary consumption per week in terms of number of fruits was 2.61±1.97, servings of green leafy vegetables were 3.44±1.92, number of eggs was 1.44±1.93, servings of meat were 1.143±1.54, and servings of a low-calorie diet were 2.23±0.88. The mean SBP was 138.2 ± 22.68 mmHg, DBP was 85.7 ± 17.59 mmHg, and RBS was 257.4 ± 79.6 mg/dL. SBP was found to be negatively correlated with meat intake and low-calorie diet consumption at a p-value <0.05, but DBP and RBS were not correlated with any dietary pattern.

Conclusion

It can be concluded that dietary interventions of intake of less meat and low-calorie foods were correlated with SBP among patients with diabetics in the 40-50 years age group. The main purpose of treating type 2 diabetes mellitus is to prevent the patients from developing complications which can be attained through proper diet compliance.

## Introduction

According to the International Diabetes Federation (IDF) Diabetes Atlas, 9th edition, globally an estimated 463 million adults aged 20-79 years were living with diabetes in 2019, and this figure is projected to rise to around 700 million by 2045. In 2019 alone, diabetes accounted for approximately 1,150,300 deaths worldwide. Over the past decade, India has experienced a rapidly growing diabetes epidemic, earning it the title of “diabetes capital of the world” [1].

Diabetes mellitus is a common metabolic disorder with multiple causes, characterized by hyperglycemia and disruptions in carbohydrate, fat, and protein metabolism due to impaired insulin secretion or resistance to insulin action. The condition is broadly classified into type 1 diabetes, type 2 diabetes, and gestational diabetes, based on etiology and clinical presentation [2]. If left untreated, diabetes can lead to serious complications, such as heart disease, stroke, kidney failure, neuropathy, blindness, diabetic foot problems, and premature death [3]. It develops either when the pancreas does not produce enough insulin, a hormone regulating blood glucose, or when the body cannot effectively use the insulin produced [4]. Diabetes is often referred to as a “silent disease” because it may show no symptoms until significant organ damage has occurred [5].

Several lifestyle-related risk factors, including poor diet, smoking, alcohol intake, overweight/obesity, and physical inactivity, increase the likelihood of developing type 2 diabetes. These factors are modifiable, and studies show effective management can reduce the risk of complications [6]. A diet rich in whole grains, fruits, vegetables, legumes, and nuts with limited refined grains, red or processed meats, sugary drinks, and alcohol can help lower diabetes risk and improve blood sugar and lipid control [7].

For individuals with type 2 diabetes or prediabetes, daily care involves maintaining appropriate diet, monitoring blood glucose, taking prescribed medication, and avoiding simple carbohydrates [8]. Increasing awareness about diabetes-related complications and improving dietary knowledge, attitudes, and practices can enhance disease management and quality of life. Healthcare providers and organizations must educate and motivate patients to adopt healthy dietary habits to aid in effective self-care and long-term disease control.

To the best of our knowledge, there is a scarcity of literature upon this topic, specifically involving the community areas of Uttarakhand. Hence, the present study was conducted to examine the correlation of dietary consumption patterns with systolic blood pressure (SBP), diastolic blood pressure (DBP), and random blood sugar (RBS) among adults with diabetes in rural and urban communities of Uttarakhand.

## Materials and methods

Study design

A quantitative research approach and a descriptive correlational research design were used in this study.

Study population

The study population were adults with diabetes who were residents of rural or urban community areas of Uttarakhand, India.

Sample size

The sample size was calculated as 159 participants. The purposive sampling technique was used in the study. The sample size was estimated using the Winpepi® program [9]. The program estimated the mean and confidence level=95%, acceptable difference=5, and assumed standard deviation (SD)=32. The final required sample size was 160. In this study, we took 159 participants into consideration for the analysis because one was found to reside outside Uttarakhand.

Eligibility criteria

The participants were individuals with a fasting blood sugar level greater than 200 mg/dL, belonging to a rural or urban community area of Uttarakhand, who provided consent to participate in the study, and understood either the Hindi or English language.

Study measures

The study's measurable outcomes were: (a) pattern of weekly dietary consumption of all food groups quantified as one bowl (25 gm) for each serving of meat, green leafy vegetables, and low-calorie diet, and the number of fruits and eggs consumed per week; (b) biophysiological parameters of SBP, DBP, and RBS; and (c) correlation between the dietary consumption pattern and SBP, DBP, and RBS. The study's data collection tool was a self-structured questionnaire regarding pattern of diet consumption status and biophysiological parameters such as SBP, DBP, and RBS. It was used to collect data from adults with diabetes in the rural or urban community areas of Uttarakhand.

The steps of data collection included the following. All participants were selected using a purposive sampling technique. Informed consent was taken from each subject recruited for the study. The questionnaire had two sections and was used to collect data between September and December 2017. The first section concerned the participants’ sociodemographic variables, whereas the second section sought outcomes related to diet consumption patterns and biophysiological parameters, such as SBP, DBP, and RBS measurements.

Ethical statement

The ethical permission for this study was taken from the Institutional Ethics Committee of the All India Institute of Medical Sciences (AIIMS), Rishikesh (Approval no: IM/RC69/2015/07). The research team maintained the confidentiality and anonymity of the participants’ records. The ethical guidelines of good clinical practices as per the Declaration of Helsinki and the Indian Council of Medical Research were followed.

Statistical analysis

Data analysis was performed using IBM SPSS Statistics for Windows, Version 23 (Released 2015; IBM Corp., Armonk, New York, United States. Age, total family members, total family income, and pattern of diet consumption as well as SBP, DBP, and RBS were recorded in the form of mean ± SD. Normality testing followed the Kolmogorov-Smrinov test, where p value>0.05 is considered normally distributed data. Correlation between pattern of diet consumption and SBP, DBP, and RBS was done by Spearman correlation test. All p values ≤0.05 were considered significant.

## Results

The data collected from 159 adults with diabetes were entered into a master sheet for tabulation and statistical processing. The data obtained were analyzed, organized, and presented under the following headings.

Distribution of the participants as per their sociodemographic variables

Out of the total study (n=159), most were female participants (n=87, 54.7%). Most of the participants (n=87; 54.7%) belonged to a joint family. However, several participants (n=47, 29.6%) were illiterate, and only a few were postgraduates (n=13; 8.2%). Most participants were homemakers (n=63; 39.6%), few earned a daily wage (n=9, 5.7%), and one was a student (0.6%). Most participants (n=110, 69.1%) lived in the rural community area of Uttarakhand (Table 1).

**Table 1:** Frequency distribution of the study sample according to the demographic variables (n=159)

S. No	Demographic variables	Frequency distribution, n (%)	
1.	Gender		
	(a) Male	72	45.3
	(b) Female	87	54.7
2.	Type of family		
	(a) Joint	87	54.7
	(b) Nuclear	51	32.1
	(c) 3^rd^ Generation	21	13.2
3.	Education		
	Postgraduate	13	8.2
	Graduate	16	10.1
	Intermediate	16	10.1
	High school	23	14.
	8^th^ Standard	27	17.0
	5^th^ Standard	17	10.7
	Illiterate	47	29.6
4.	Occupation		
	Employed	27	17.0
	Unemployed	20	12.6
	Student	1	0.6
	Retired	18	11.3
	Homemaker	63	39.6
	Self-employed	21	13.2
	Daily wager	9	5.7
5.	Residence		
	(a) Rural	110	69.18
	(b) Urban	49	30.8

The study population had a mean age of 53.4 ± 1.23, whereas the mean ± SD family income was INR 19820.30 ± 17.85. The average total family members was 5.4088 ± 2.60 (Table 2).

**Table 2:** Mean of the selected sociodemographic variables (n=159)

Demographic variables	Mean	Standard deviation
Age	53.4	1.23
Family income (INR per month)	19820.30	17652.85
Total family members	5.4088	2.60

Pattern of diet consumption and SBP, DBP, and RBS

Responses to the structured questionnaire on the dietary consumption indicated that the mean consumption per week in terms of number of fruits was 2.61 ± 1.97, servings of green leafy vegetables were 3.44 ± 1.92, number of eggs was 1.44 ± 1.54, servings of meat was 1.14 ± 1.54, and servings of low-calorie intake 2.23 ± 0.88 (Table 3).

**Table 3:** Pattern of diet consumption (n=159)

Diet consumption	Mean	Standard deviation
Number of fruits/week	2.61	1.973
Servings of green leafy vegetables/week	3.44	1.92
Number of eggs/week	1.44	1.93
Servings of meat/week	1.14	1.54
Servings of low-calorie intake/week	2.23	0.88

The mean SBP of the participants was 138.2 ± 22.68 mmHg, DBP was 85.7 ± 17.59 mmHg, and RBS was 257.4 ± 79.6 mg/dL (Table 4).

**Table 4:** Mean values for blood pressure and sugar levels (n=159)

Blood parameters	Mean	Standard deviation
Systolic blood pressure (SBP; mmHg)	138.17	22.6
Diastolic blood pressure (DBP; mmHg)	85.68	17.59
Random blood sugar (RBS; mg/dL)	257.48	79.6

Correlation between the pattern of diet consumption and mean SBP, DBP, and RBS

There was a significant but weak positive correlation (r value=0.016) between mean SBP and consumption of meat (p=0.042), suggesting that increased consumption of meat would further increase SBP (Table 5).

**Table 5:** Correlation between mean systolic blood pressure (SBP) and diet patterns (n=159) ^*^p≤0.05 is considered significant.

Pattern of diet consumption	r-value	p-value
Fruits/week	0.05	0.950
Green leafy vegetables/week	-0.144	0.069
Eggs/week	0.016	0.840
Meat/week	0.016	0.042*
Low calorie intake/week	-0.164	0.039*

Similarly a weak negative correlation (r value=-0.169) was found between mean SBP and consumption of a low-calorie diet (p=0.039), suggesting an increased intake of low-calorie diet could reduce SBP. However, SBP did not correlate with other dietary consumption patterns among adults with diabetes.

There was no significant correlation found between DBP and pattern of dietary consumption among adults with diabetes (Table 6).

**Table 6:** Correlation between mean diastolic blood pressure (DBP) and pattern of diet consumption (n=159)

Pattern of diet consumption	r-value	p-value
Fruits/week	-0.006	0.942
Green leafy vegetables/week	-0.013	0.868
Eggs/week	0.052	0.517
Meat/week	0.085	0.288
Low-calorie intake/week	-0.057	0.449

There was no significant correlation found between RBS and pattern of dietary consumption among adults with diabetes (Table 7).

**Table 7:** Correlation between mean random blood sugar (RBS) and pattern of diet consumption (n=159)

Pattern of diet consumption	r-value	p-value
Fruits/week	0.26	0.741
Green leafy vegetables/week	0.019	0.816
Eggs/week	0.020	0.805
Meat/week	0.020	0.798
Low-calorie intake/week	0.060	0.449

## Discussion

The present study included 159 individuals with diabetes, with most being women. The total dietary consumption of participants indicated that the mean weekly consumption in terms of number of fruits was 2.61 ± 1.97, servings of green leafy vegetables were 3.44 ± 1.92, number of eggs was 1.44 ± 1.54, servings of meat were 1.14 ± 1.54, and servings of low-calorie intake were 2.23 ± 0.88. The mean SBP of the participants was 138.2 ± 22.68 mmHg, DBP was 85.7 ± 17.59 mmHg, and RBS was 257.4 ± 79.6 mg/dL. The weak positive correlation between mean SBP and the consumption of meat suggested that increased consumption of meat would further increase SBP. Similarly, a weak negative correlation was found between mean SBP and a low-calorie diet, suggesting that increased intake of a low-calorie diet would reduce SBP. DBP was not correlated with RBS and diet consumption.

Globally, type 2 diabetes mellitus is the most common form of the disease and rising rates in developing countries are linked to dietary habits and sedentary lifestyles. In Saudi Arabia, growing portion sizes and a highly inactive lifestyle have greatly increased obesity rates, partly because of the convenience of fast food, which contributes to the country’s alarming diabetes statistics [10].

The World Health Organization estimates that about 200 million people worldwide have diabetes, and this number could double by 2030. In a study by Rahmanian et al. [11], only 10.4% of the 700 participants had a family history of diabetes. Another investigation by Raikar et al. [12] in Mumbai’s urban slums found a 27.4% positive family history. Diet plays a key role in controlling blood glucose. Foods low in calories and glycemic index and high in fiber are strongly recommended. Insoluble fiber supports bowel movement, whereas soluble fiber helps lower cholesterol [13].

Backman [14] highlighted that nutritional knowledge significantly affects eating behaviors, consistent with the current findings. Similarly, Savoca and Miller [15] observed that patients’ food choices and habits are shaped by their understanding of the dietary guidelines for diabetes. In this study, a weak negative correlation was found between low-calorie intake and SBP, aligning with another study that reported a significant positive relationship between knowledge of diabetic diets and awareness of calorie needs (p<0.05) [16]. Overall, the evidence suggests that understanding diabetic diets is essential for improving eating behaviors.

Research indicates that diets rich in starch and monounsaturated fats can enhance insulin sensitivity, and dietary management is a first-line strategy for controlling dyslipidemia in patients with diabetes [17]. Several interventional studies recommend nutrition therapy and lifestyle changes as initial treatments for dyslipidemia. Because carbohydrate intake directly affects post-meal glucose levels, it is the primary macronutrient of concern in glycemic management [18]. Food choices and energy balance also influence body weight, blood pressure, and lipid levels. Health professionals can help patients meet their health goals by providing personalized nutrition plans and ongoing support [19].

Dietary habits are critical to cardiovascular and metabolic health [20]. The Mediterranean diet, rich in fruits, vegetables, fish, and olive oil, has shown many health benefits, including improved glucose metabolism and reduced risk of type 2 diabetes, obesity, and cardiovascular disease [21,22]. High nutrition knowledge has also been associated with better adherence to dietary recommendations and more effective self-management. In Indonesia, however, a study found a preference for high-fat foods among diabetic patients, increasing cardiovascular risk [23]. Skipping breakfast has become more common over the past two decades across all age groups [24].

A prudent dietary pattern emphasizes fish, poultry, vegetables, and fruits, whereas a “Western country’s” pattern includes more processed and red meat, chips, dairy products, refined grains, sweets, and desserts, patterns previously linked to higher type 2 diabetes risk. The glycemic index measures the rise in blood glucose after consuming a given carbohydrate compared to white bread or pure glucose, and the glycemic load reflects both the quality and quantity of the carbohydrates consumed [25].

Improved dietary knowledge, attitudes, and practices are integral to comprehensive diabetes care. Although the prevalence of diabetes is high in Gulf countries, many patients still lack awareness of diet’s role in disease management [26]. Historical evidence also supports the link between food availability and diabetes outcomes: during World Wars I and II, death rates due to diabetes dropped in countries experiencing food shortages. In Germany, the rate fell from 23.1 per 100,000 in 1914 to 10.9 in 1919. In contrast, no such change occurred in nations without food scarcity, such as Japan and the United States [27].

Numerous studies have linked high carbohydrate and fat intake to type 2 diabetes [28]. Ludwig’s long-term study of over 500 ethnically-diverse schoolchildren found that each additional daily serving of soda increased obesity risk, even after accounting for diet, demographics, and lifestyle factors [29]. Another study examined dietary components, such as fat, fiber, and sucrose, and found no significant associations with diabetes risk after adjustment [30]. More recent evidence connects consumption of sugar-sweetened beverages, especially those high in high-fructose corn syrup, to obesity and diabetes by elevating blood glucose and body mass index [31]. Conversely, greater fruit and vegetable intake, rich in nutrients, fiber, and antioxidants, has been shown to lower the risk of type 2 diabetes, consistent with the present findings [32]. Overall, reviews emphasize the importance of diabetes education, including dietary management, to promote self-care and improve quality of life [33].

The study’s strengths include its focus on a relevant public health issue, adequate sample size, various age groups assessed. and inclusion of both rural and urban participants, which enhances generalizability. The use of correlation analysis and descriptive statistics is appropriate for the research design. A correlation was conducted with both blood pressure and RBS that was not manipulated by participants' capability for recall. Limitations include study design, descriptive study, which could have been an experimental design. Also, a control group could have been included. Others limitations could be the non-random sampling method and the weak correlations found, which limit the strength of causal inference.

## Conclusions

The present study revealed that green leafy vegetables were the most commonly consumed food in the weekly diet of older adults. However, blood pressure and RBS levels remained elevated in this community, indicating a need for targeted health interventions.

Dietary modifications and lifestyle changes are essential strategies for managing blood glucose and blood pressure levels, particularly among individuals aged 40-50 years. Ensuring cultural, familial, and community diversity among participants can strengthen the effectiveness of dietary management.

Additionally, patient awareness and education regarding disease and diet patterns play a crucial role in prevention and control. Healthcare providers should actively encourage and guide patients to adopt healthier nutritional practices. Future research should focus on well-structured dietary interventional studies aimed at preventing the onset of diabetes and its related complications.

## Appendices

**Table 8:** Self-structured questionnaire administered to the study participants

	Section-A	
S. No	Demographic variables	Yes/No
1.	Age (in years)	
2.	Gender	
A	Male	
B	Female	
3.	Type of family	
A	Joint	
B	Nuclear	
C	3^rd^ Generation	
4.	Education	
A	Postgraduate	
B	Graduate	
C	Intermediate	
D	High school	
E	8^th^ Standard	
F	5^th^ Standard	
G	Illiterate	
5.	Occupation	
A	Employed	
B	Unemployed	
C	Student	
D	Retired	
E	Homemaker	
F	Self-employed	
G	Daily wager	
6.	Residence	
A	Rural	
B	Urban	
7.	Family income (INR per month)	
8.	Total family members	
	Section-B	
1	Pattern of dietary consumption	
A	Number of fruits/week	
B	Servings of green leafy vegetables/week	
C	Number of eggs/week	
D	Servings of meat/week	
E	Servings of low-calorie intake/week	
2	Biophysiological parameters	
A	Systolic blood pressure	
B	Diastolic blood pressure	
C	Random blood sugar	
